# Assessing the progression of systemic sclerosis by monitoring the tissue optic axis using PS-OCT

**DOI:** 10.1038/s41598-020-59330-7

**Published:** 2020-02-13

**Authors:** David C. Adams, Margit V. Szabari, David Lagares, Andrew F. McCrossan, Lida P. Hariri, Andrew M. Tager, Melissa J. Suter

**Affiliations:** 1 Division of Pulmonary and Critical Care Medicine, Massachusetts General Hospital, 40 Blossom Street, Boston, Massachusetts 02114 USA; 2 Department of Pathology, Massachusetts General Hospital, Harvard Medical School, Boston, MA 02114 USA; 3 Wellman Center for Photomedicine, Massachusetts General Hospital, 50 Blossom Street, Boston, Massachusetts 02114 USA; 4 Harvard Medical School, 25 Shattuck Street, Boston, Massachusetts 02115 USA

**Keywords:** Skin diseases, Experimental models of disease, Translational research, Skin manifestations, Imaging and sensing

## Abstract

The clinical assessment of fibrosis is critical to the diagnosis and management of patients with systemic sclerosis. Current clinical standards for patient assessment is to use skin fibrosis as an indicator of organ involvement, though this approach is highly subjective and relies on manual palpation. The development of a new method for accurately quantifying collagen content may therefore significantly improve the accuracy of the traditional skin score in patients with systemic sclerosis and may additionally aid in the monitoring of anti-fibrotic therapies in clinical practice. Polarization-sensitive optical coherence tomography (PS-OCT) is a high-speed volumetric imaging modality that can be used to assess birefringent tissues including collagen. In this work we demonstrate a novel computational approach using PS-OCT for the assessment of fibrosis. This approach, based on the measured distribution of optic axis values associated with a given volume of collagen orientation, characterizes fibrotic changes independently from the depth of the region of interest in the tissue. This approach has the potential to accurately quantify collagen content and orientation faster and more robustly compared to traditional PS-OCT metrics. We investigate the viability of this approach for assessing the development of fibrosis in a bleomycin induced skin fibrosis mouse model.

## Introduction

Systemic sclerosis (SSc) is an immune mediated disease with an overall incidence rate of approximately 20 per million per year in the adult population of the United States^1^. In this rare, but severe disease with high morbidity and mortality^2^, deposition of extracellular matrix proteins, predominately collagen, occurs^3^. This process termed fibrosis, can affect the skin, the lung, and the gastrointestinal system, and other organs and can ultimately lead to organ failure^4^. Skin fibrosis is a key component of SSc and is often used to predict internal organ involvement^5^, patient survival^6^ and to assess disease severity^7^. Histologic evaluation of skin fibrosis^8^ revealed that as fibrosis progresses, collagen fibers become more organized and densely packed than the collagen fibers in healthy skin.

The current clinical standard for assessing skin involvement relies on the modified Rodnan skin score (mRRS) test. This test involves manual palpation of the skin by the physician^9,10^, and is therefore highly subjective resulting in high intra-observer and inter-observer variability^11^. The mRSS test aims to assess the skin in a noninvasive way, but it is unclear if the method differentiates between skin thickness, hardness or tethering^12^ all of which are influenced by late stage fibrosis. It does not however, assess the less subtle morphological changes including collagen fiber orientation that provide a more sensitive measure of disease development^8^. Thus, there is a significant demand for a new tool to objectively assess the skin on a microscopic level over a large field of view without the need for invasive tissue biopsy.

Nonlinear optical microscopy techniques, such as second harmonic generation, are able to achieve remarkable visualization of collagen with endogenous contrast^13^, but limited field of view and scanning capabilities make them ill-suited for clinical use. Optical Coherence Tomography (OCT) is considered a high-resolution optical analog to ultrasound, which has been used to image dermal thickness for the purposes of SSc diagnosis and treatment for a number of decades^7^. We believe that OCT and its extension, polarization sensitive OCT (PS-OCT) are better suited for clinical imaging of SSc. OCT is a volumetric, high-speed imaging modality capable of providing depth-resolved assessment of centimeter-scale volumes in seconds, and with resolution approaching that of histology (~10 microns)^14,15^. PS-OCT extends the utility of OCT with endogenous contrast for birefringent tissue such as collagen^16–18^, making it ideally suited to the clinical assessment of fibrosis of the dermis as well as that of other organ systems.

Other groups have used structural OCT to assess skin fibrosis in SSc patients and have found decreased scattering intensity notably at the dermal-epidermal junction in SSc patients compared to healthy volunteers^15,19–21^. To date, only a handful of studies involving PS-OCT imaging of dermal fibrosis have been conducted^22–25^, featuring measurements of sample birefringence parameters such as phase retardance and degree of polarization (DOP) for characterizing collagen content. Although these studies have demonstrated the potential of PS-OCT for characterizing collagen, arguably one of the most important drawbacks of the approaches employed is the strong dependence on the dermal depth region selected for the assessment. This dependence, coupled with the variations in dermal thicknesses between normal and fibrotic tissue as well as on different regions of the body^26^, makes it difficult to assess the collagen structure of the dermis rapidly and accurately without the need for careful region of interest (ROI) selection within the dermis.

In this work we demonstrate a novel approach of estimating collagen fiber density and orientation by assessing the entropy of optic axis (OA) values measured with PS-OCT. This approach is designed to provide a quantitative measure of the morphological changes to collagen fibers in fibrosis^8^, and has the potential to offer faster and more robust analysis compared to traditional PS-OCT metrics. We demonstrate the potential of this approach in assessing scar tissue versus normal skin in a human volunteer and conduct a preclinical study assessing fibrosis development in a mouse model of scleroderma.

## Results

### OA entropy

In many types of biological tissue containing highly ordered collagen, such as muscle or skin tissue, birefringence intuitively suggests an association of the optic axis (OA) with collagen fiber orientation. OA is a characteristic derived from the PS-OCT imaging (see Methods). Entropy or heterogeneity, in the context of image analysis, is a histogram-based measurement defined as:

1 $$H=-\sum {p}_{i}{{\rm{\log }}}_{2}({p}_{i})$$

**Figure 1:**
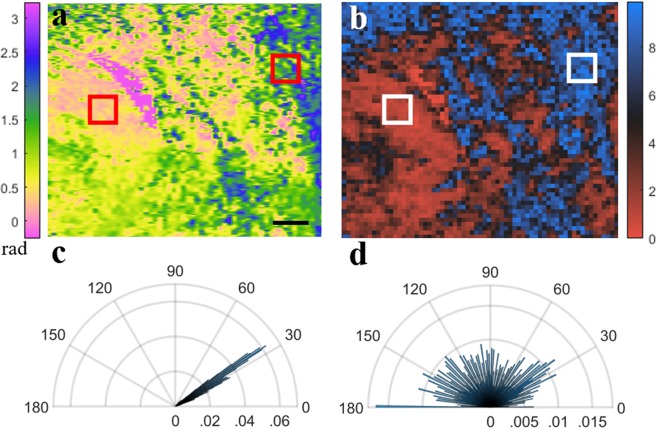
Example demonstrating OA entropy measurement using data obtained from skin. (**a**) *en face* OA map from approximately 150 *μ*m 2 D planes into the dermis, and (**b**) corresponding OA entropy map, using 100 *μ*m^2^ 3 D blocks for entropy calculation. (**c**,**d**) The polar histograms for a low entropy (left highlighted region on panel B) and high entropy (right highlighted region on panel B), respectively. Scale bar, 1 mm.

Where *p*_*i*_ is the count associated with the number of occurrences in a given histogram bin *i*. In the analysis presented here, the binned value is the OA angle in the Q-U plane on the Poincare sphere, and the ROI per calculation is a voxel of biologically appropriate dimensions. OA entropy would inform us the heterogeneity of collagen fiber orientation via optic axis values measured by PS-OCT. This approach has the benefits of being intuitively associated with the arrangement of collagen fibers within the analyzed voxel, and of being both easily implemented and computationally efficient. Figure 1 demonstrates our approach using data obtained from the dorsal skin of a mouse. A single *en face* 2 D plane of OA measurements from approximately 150 *μ*m into the dermis (Fig. 1a) is converted to an OA entropy 3 D map using 100  ×  100 *μ*m^2^ block dimensions per calculation (Fig. 1b). Figure 1(c,d) show the polar histograms associated with a low entropy (highly organized, packed collagen) (Fig. 1c) and high entropy (less organized collagen fibers) (Fig. 1d). 256 bins were used for this and for all other entropy measurements reported in this work.

### Human Scar

**Figure 2:**
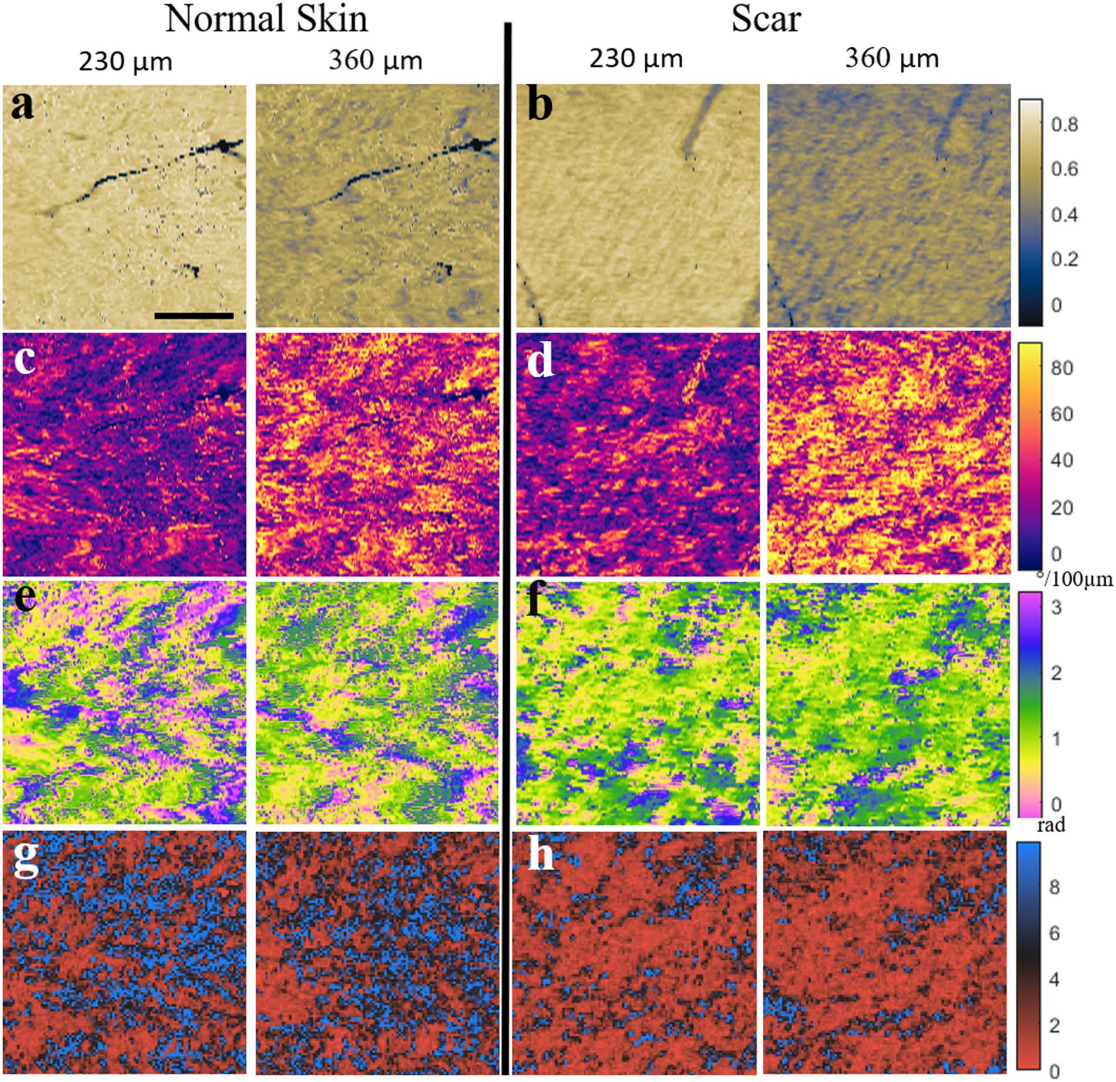
Comparison between normal and fibrotic human forearm skin at two different depths (230 *μ*m and 360 *μ*m) for (**a**,**b**) DOP, (**c**,**d**) phase retardance, (**e**,**f**) OA, and (**g**,**h**) OA entropy. A strong variation with depth can be observed for the first two measurements, and a much milder variation for the last two. Scale bar, 1 mm.

To test our new dermal depth independent imaging approach, we assessed normal and normotropic scar tissue in a human volunteer. The goal of developing this new approach to evaluate fibrosis with PS-OCT was to mitigate the dependence on depth that is typically observed with traditional PS-OCT measurements. This dependence is clearly demonstrated in Fig. 2, in which we imaged 3x3 mm^2^ block of both normal and normotrophic scar tissue from a human volunteer. The normotrophic scar tissue was located on the back of the forearm and the normal skin was imaged at the same location on the opposite arm in order to provide the best comparison. *En face* images from two different depths plane in the dermis (230 *μ*m and 360 *μ*m, in alternating columns) demonstrate the variation with depth for both the degree of polarization (DOP) (Fig. 2a,b) and the local retardance (Fig. 2c,d), both of those metrics used in previous studies^22–25^ to image skin fibrosis with PS-OCT. In contrast, the collagen orientations as measured with the OA appear highly consistent with depth (Fig. 2e,f). The last row in the figure (Fig. 2g,h) shows the OA entropy associated with the OA measurements, again using 100 × 100 *μ*m^2^ 3 D block dimensions for each of the *en face* planes. These results demonstrate both the consistency between depths and the notable decrease in OA entropy associated within the normotrophic scar tissue compared to the normal dermis.

**Figure 3:**
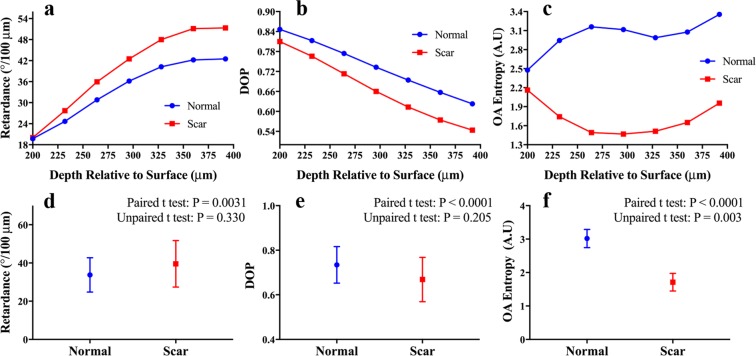
(**a**–**c**) Measurement versus depth over a broader range of depths for the data depicted in Fig. 2. (**a**) Retardance, (**b**) DOP, and (**c**) OA entropy. (**d**–**f**) Corresponding column graphs for the three measurements of (**a**–**c**). The error bars indicate the standard deviation for each set.

The relationship between dermal depth location and DOP, phase retardance, and OA entropy is demonstrated for a broader range of depths in Fig. 3(a–c). The first measurement was taken 200 *μ*m from the skin surface in order to ensure measurements were obtained in the dermis rather than the epidermis^27^. Each data point represents the total average obtained from the area of skin samples indicated in Fig. 2. The dependence on depth between the three measurements can be further quantified by comparing the paired (depth-matched) and unpaired (not depth-matched) relationship between the normal and normotrophic skin samples (Fig. 3d–f). These results demonstrate that when the measurements are compared at corresponding depths each of the three measurements exhibit statistically significant differences between normal and scar tissue (P = 0.0031 for retardance, P < 0.0001 for DOP and OA entropy), whereas when depth matching is not taken into account only the OA entropy measurement is significantly different (P = 0.003).

### Bleomycin mouse model of skin fibrosis

**Figure 4:**
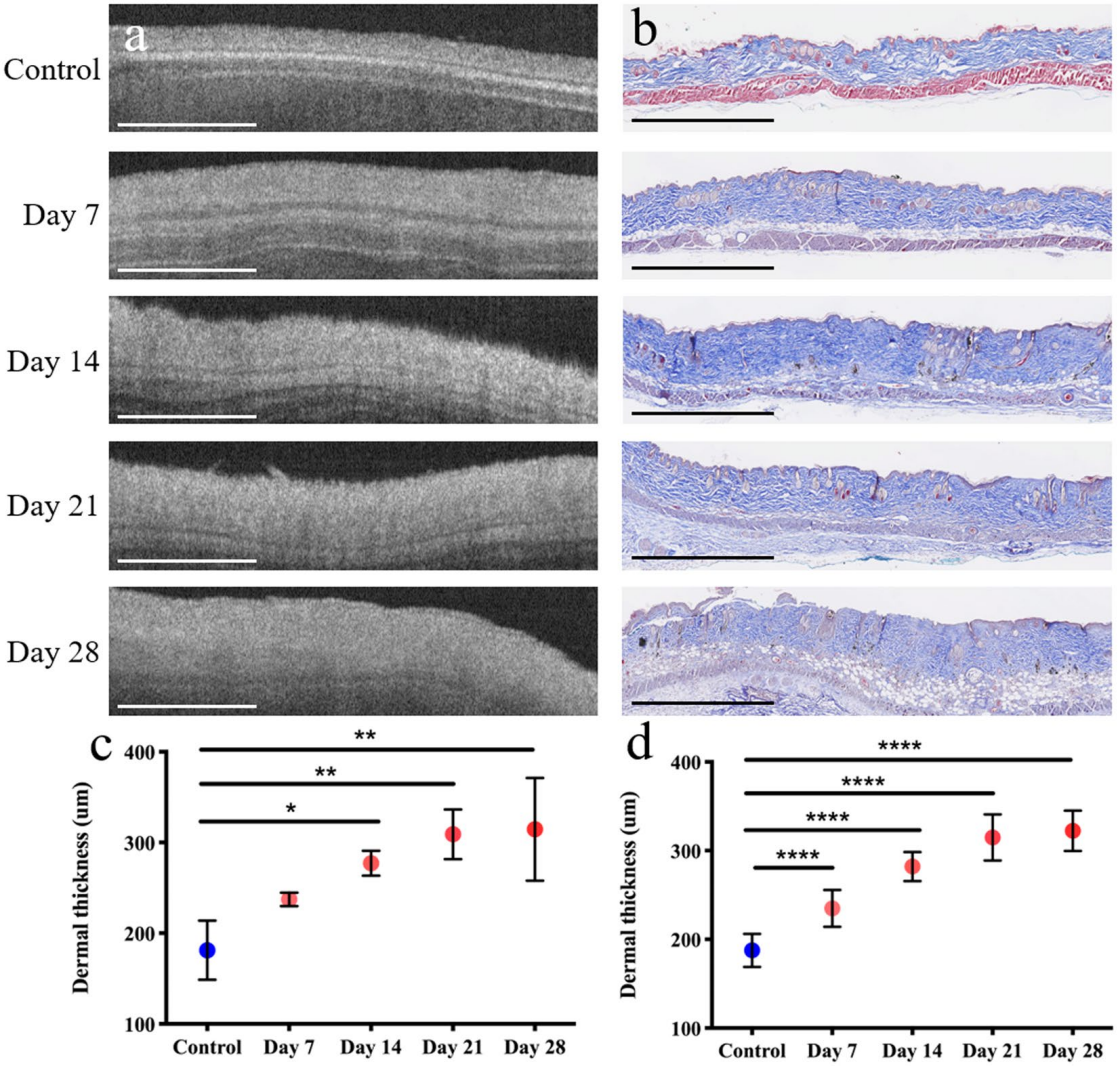
Cross-sections of mouse skin for PBS-injected (control) samples and bleomycin-injected samples (day 7 - day 28). (**a**) OCT images and (**b**) Masson’s Trichrome stained histology. Similar increases in dermal thickness were observed in measurements performed in (**c**) OCT images and (**d**) histology. *P < 0.05, **P < 0.01, ***P < 0.001, ****P < 0.0001. Scale bars, 1 mm.

**Figure 5:**
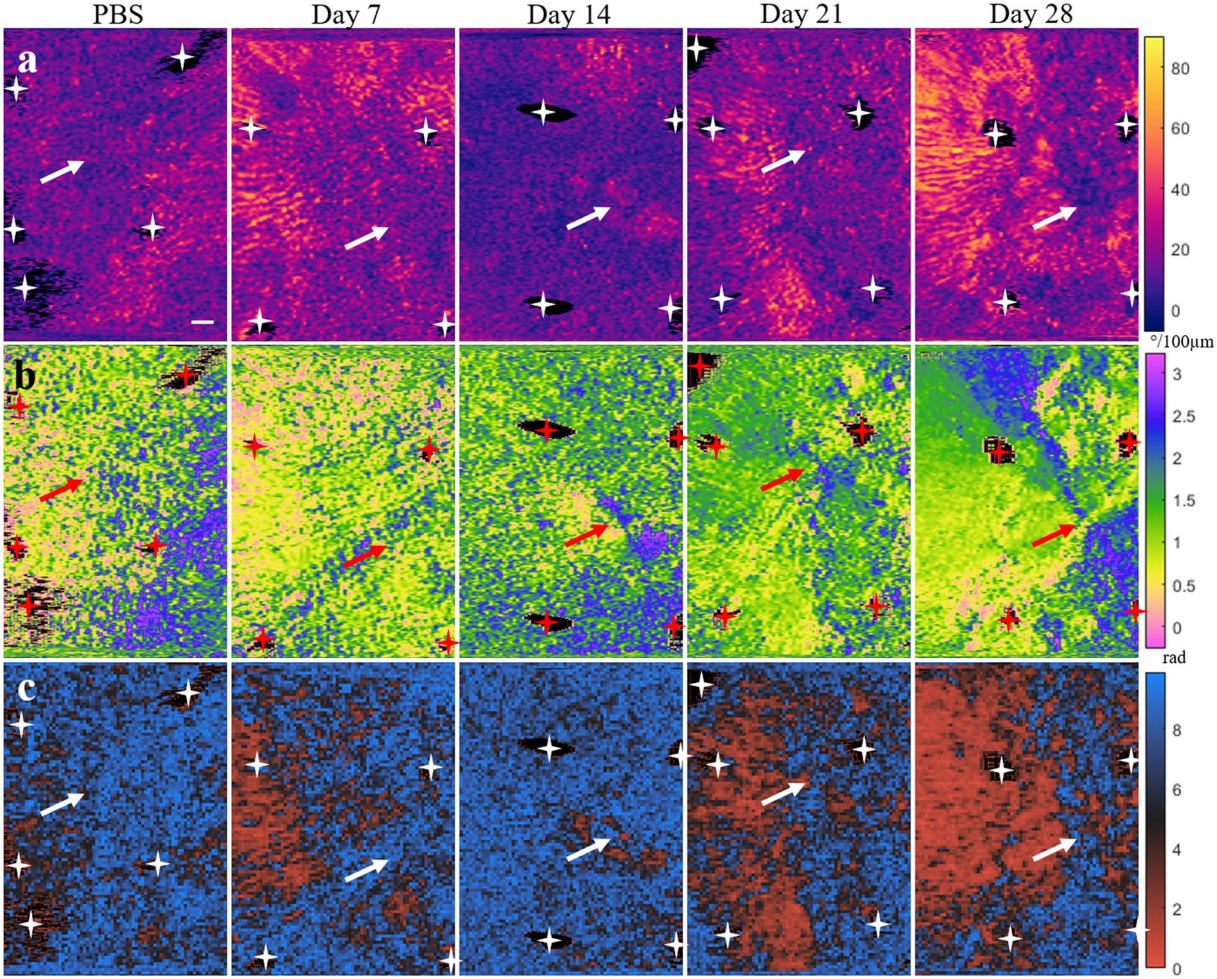
*En face* images of mouse skin samples for both PBS and bleomycin-treated skin (day 7- day 28). (**a**) Retardance, (**b**) OA, and (**c**) OA entropy. Both retardance and OA images were obtained from 150 *μ*m into the dermis, while OA entropy was assessed volumetrically from 120 to 190 *μ*m in depth. Arrows indicate approximate injection site. Stars indicate regions stained with tissue ink for registration purposes that were excluded from analyses. Scale bar, 1 mm.

For a more comprehensive approach, we applied our technique to the study of skin fibrosis in a mouse model. In this study we employed a bleomycin induced model for skin fibrosis^28,29^, imaging both control and fibrotic mice skin in order to characterize disease progression (see Methods). In total, 15 samples were imaged in this study: 3 control (PBS-injected) and 3 obtained from each of the 4 endpoints for bleomycin injections (7, 14, 21, or 28 days of injections). Figure 4(a,b) shows representative dermal cross-sections from both histology and from our OCT imaging. Similar increases in dermal thickness were observed in measurements obtained from both OCT images and histology (Fig. 5c,d). These results, comparable to other results that have been obtained in bleomycin studies^30^, demonstrate that fibrosis developed in the anticipated manner following repeated bleomycin injections.

**Figure 6:**
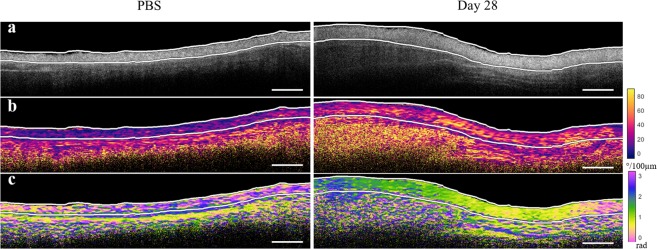
Representative cross-sections for the control and bleomycin-treated day 28 skin scans shown in Fig. 5. (**a**) Structural, (**b**) retardance, and (**c**) OA cross-sections. The white lines in each of the images denote the boundaries of the dermis. Scale bars, 1 mm.

Representative *en face* images for each of the bleomycin endpoints, as well as the controls, are depicted in Fig. 5. The phase retardance (Fig. 5a) and OA (Fig. 5b) images were obtained from 150 *μ*m 2 D plane into the dermis. The OA entropy (Fig. 5c) was calculated using 3 D voxels 100x100 *μ*m^2^ in transverse area and extending from 120 *μ*m into the dermis to 190 *μ*m into the dermis (70 *μ*m thick). Regions that are color-coded black and indicated with stars in the images indicate where ink markings were placed during the study for registration purposes and were excluded from all analyses. The approximate location of the injection site is indicated with arrows based on the structural OCT imaging. Pronounced differences in collagen architecture between normal and fibrotic dermises are observed in the OA images (Fig. 5b) for the control and day 28 skin samples. While the normal collagen appears loosely, heterogeneously oriented in the PBS-injected skin, a uniform, radial pattern has clearly emerged by day 28 in the bleomycin-injected skin. Furthermore, this pattern appears to originate from the site of the injection itself, suggesting that this location could even be determined *a posteriori* by analyzing the OA data. Representative cross-sectional images for both the control and day 28 skin samples are shown in Fig. 6.

**Figure 7:**
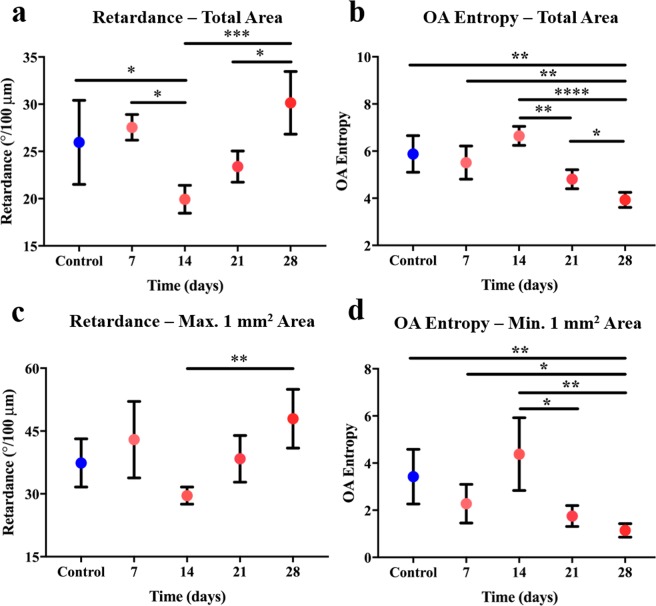
Column graphs representing average values from PBS-injected (control) and bleomycin-injected (day 7- day 28) samples for (**a**) retardance (total imaged area), (**b**) OA entropy (total imaged area), (**c**) retardance (1 mm^2^ area with largest average), and (**d**) OA entropy (1 mm^2^ area with smallest average). *P < 0.05, **P < 0.01, ***P < 0.001, ****P < 0.0001.

Quantitative results of the study are presented in Fig. 7. Each data point represents the average value obtained from the 3 corresponding samples, with the standard deviation indicated with error bars. Both the retardance (Fig. 7a,c) and the OA entropy (Fig. 7b,d) values obtained per imaged sample were calculated over the same 120 *μ*m to 190 *μ*m thickness used to generate the OA entropy images depicted in Fig. 5(c). Figures 7(a,b) were obtained by averaging the entire 10x14 mm^2^ imaging region per sample. Because it is possible that this imaging region included skin unaffected by fibrosis, we also compared 1 mm^2^ areas from each sample that contained the highest retardance (Fig. 7c), and 1 mm^2^ areas with the smallest OA entropy (Fig. 7d). The fact that the overall trend in the data is maintained between Fig. 7(a–d) suggests that the ROI was not a determining factor in our analysis.

## Discussion

The clinical assessment of skin fibrosis in individuals with SSc is highly subjective and prone in intra- and inter- observer variability, yet is routinely used as a predictor of disease progression and internal organ involvement. The goal of this project was to develop and validate a non-invasive imaging approach to provide objective, straightforward and efficient assessment of skin fibrosis in the clinical setting. In this study, we used PS-OCT to image birefringence in fibrotic and healthy skin and demonstrated that OA and OA entropy provide valuable insight into the assessment of fibrosis. When compared to previously reported methods we demonstrated that our novel volumetric assessment of fibrosis with OA entropy is an invaluable tool for characterizing skin fibrosis objectively, independently of dermal depth, and without the need of careful ROI selection. We tested our method in a mouse model of Sc and demonstrated that we could successfully monitor the disease progression over time. Importantly, we were able to differentiate between early and late fibrotic changes in the mouse skin using the OA and OA entropy parameters calculated from the PS-OCT imaging.

Previous work using PS-OCT imaging of dermal fibrosis^22–25^ was characterized by measurements of sample polarization parameters such as phase retardance and degree of polarization (DOP) for characterizing collagen quantity. The application of both metrics is clinically limited due to their strong dependence on the dermal depth region selected for the assessment. This dependence is most like due to changes in collagen distribution^31^ and/or the decrease in signal to noise ratio (SNR)^32^ with increasing dermal depth. In addition, the variation of dermal thickness in different locations and in different stages of fibrotic disease development make it difficult to interpret these metrics in the clinical setting. Our approach is instead based on the entropy of sample OA vectors, which have a direct relationship with the orientation of the collagen fibers and is found to be minimally impacted by dermal thickness or depth ROI. A potential disadvantage associated with this approach is the fact that an additional processing step is required to obtain the results. However, we believe this disadvantage is significantly offset by the ease with which this approach may be automated in order to minimize the need for manual segmentation.

Based on our preliminary results on human skin, we anticipated that low OA entropy would characterize the fibrotic skin in our SSc mouse model, as highly ordered collagen deposited into the tissue. We expected to observe a continual decrease in OA entropy during the course of the study as the fibrosis progressed and more densely packed and highly oriented collagen deposited in the dermis. Contrary to this, we observed an increase in OA entropy on day 14 (Fig. 7) although the dermal thickness continuously increased during the timeline of the study (Fig. 4). Similar results have been observed in other bleomycin models where collagen assay was used for fibrotic assessment^33^, providing support for the validity of our technique. We believe that these results likely differentiate between the early stages (day 14) of fibrosis development, which is characterized by the deposition of randomly arranged disorganized collagen fibrils, and later stages (day 21 and 28) where the collagen appears highly organized and tightly packed reflected in the decreasing OA entropy results^34^.

In summary, we have developed a technique for quantitatively assessing fibrosis. This technique, based on the entropy of OA measurements obtained from within the dermis, exhibits only a mild dependence on the depth at which the measurement is obtained compared to traditional PS-OCT measurements. It was derived from the observation that collagen deposited in response to injury and in many types of fibrotic diseases exhibits a more uniformly oriented architecture compared to normal collagen^8^. We believe that this approach could replace current subjective clinical approaches for assessing fibrosis by offering the ability to provide large-scale quantitative assessments. Fiber optic PS-OCT has been performed in intracoronary^35^, gastrointestinal^36^ and other endoscopic applications^37^. Though our study focused on the assessment of dermal fibrosis, PS-OCT imaging and OA entropy analysis could open the opportunity to objectively follow and compare disease progression, treatment and management of other organs within the body.

## Methods

The studies were approved by Partners Healthcare Institutional Review Board (IRB) at Massachusetts General Hospital, and informed consent was obtained from all subjects. The animal experiments were approved by Institutional Animal Care and Use Committee (IACUC) at Massachusetts General Hospital. The studies were carried out in accordance with the relevant guidelines and regulations.

### PS-OCT system

All of the PS-OCT data presented here was collected using a custom-built fiber-based swept-source OCT system centered at 1310 nm with a depth range of approximately 1 cm, an axial resolution (in tissue) of approximately 7 *μ*m, and an A-line acquisition rate of 66 kHz. An electro-optic modulator in the sample arm was used to alternate the input polarization state between A-lines in order to avoid ambiguities in sample birefringence. A benchtop microscope with a telecentric lens (Thorlabs LSM03) was used to image the samples, and the lateral and transverse scanning were achieved using galvonometric mirrors. Additional details regarding the technical specifications of this system have been previously described^38^.

### Image processing

In a standard configuration for a PS-OCT system in which two orthogonal polarization states of light are used to probe the sample, both the local retardance and the OA of the sample can be obtained through a straightforward geometric solution^18^, while the DOP can be calculated from the ratio of the norm of unfiltered to that of filtered Stokes vectors^39^. For our PS-OCT data processing, a gaussian filter with a width of 28 *μ*m at 1 standard deviation was applied to the Stokes data, and a depth offset of 20 *μ*m was used for the local birefringence calculation.

Because a fiber-based PS-OCT system was used in this study, the measured OA of the sample was rotated in the Poincare sphere by the unknown birefringence of the single mode fiber in the path from the source to the sample and from the sample to the detection arm. Although methods for compensating for this exist^40–42^, it was unnecessary in this study due to the fact that we were not interested in the OA vectors themselves, but rather their combined entropy. Instead, we used a least-squares based approach to find the plane in the Poincare sphere that contained our measured OA sample vectors and converted them to scalar angles in the plane relative to an arbitrary, fixed axis:

2 $$\theta =\frac{1}{2}{\rm{atan2}}({S}_{2},{S}_{1})\quad 0\le \theta \le \pi $$

Where *S*_1,2_ are the components of the OA vector along the two axes that define the plane. For our entropy-based assessment, our sample volume was divided into non-overlapping voxels of equal dimensions and the entropy of OA angles within each voxel averaged over the entire volume to produce a single measurement that could be compared between samples. All data was processed using Matlab 2015.

Dermal thickness measurement was performed on cross-sectional OCT images. We used ImageJ tool to randomly and manually measure approximately twenty locations per skin samples. Dermal thickness measurement was performed on one histological slide per skin sample. We used NanoZoomer Digital Pathology tool to randomly and manually measure approximately ten locations per skin samples. The dermal thickness measurements on OCT images and histological samples were then averaged, and the mean and standard deviation were plotted using GraphPad Prism.

### Mouse model of skin fibrosis

In this study we used a mouse model of skin fibrosis involving subcutaneous injections of the fibrosis-inducing drug bleomycin, which has been used extensively to study the disease pathogenesis^28,29^. The study details are as follows. Bleomycin was dissolved in phosphate buffered saline (PBS) at 10 *μ*g/ml and sterilized by filtration. Bleomycin or PBS (100 *μ*l from stock solution) was injected daily into two locations on the shaved back of wild type (C57BL/6) mice^28,43^. To investigate scleroderma over a range of severities, these injections were performed daily for 7, 14, 21, or 28 days. At the pre-determined endpoint (7, 14, 21, or 28 days of injections) the following procedure was performed: first, a 10 mm  ×  14 mm region in the neighborhood of each injection site was imaged with our PS-OCT system at both locations on the back of the mouse. Before the imaging, the mice were sacrificed, and the skin of the mouse was inked with tissue markers to assure orientation during the image analysis and histology. After the imaging, the imaged dermis was removed and fixed in formalin. For histology, multiple 5 *μ*m thick sections were cut from each dermal sample and stained with Masson’s Trichrome (collagen stained blue).

### Statistics

Statistical analyses were performed using Graphpad Prism. PS-OCT data from human skin were compared using Student’s t-tests, using paired matching for depth-dependent statistics and unpaired matching for depth-independent statistics. Measurements obtained for mouse study, including skin thickness in histology and OCT images as well as PS-OCT metrics, were compared using one-way ANOVA with Tukey’s multiple comparison post-test to assess differences between individual data points. Results were considered significant for P < 0.05.

## References

[1] Mayes MD (2003). Scleroderma epidemiology. Rheum. Dis. Clin. North Am..

[2] Denton CP, Khanna D (2017). Systemic sclerosis. Lancet.

[3] Santos A, Lagares D (2018). Matrix stiffness: the conductor of organ fibrosis. Curr. Rheumatol. Rep..

[4] Ho YY, Lagares D, Tager AM, Kapoor M (2014). Fibrosis-a lethal component of systemic sclerosis. Nat. Rev. Rheumatol..

[5] Clements PJ (1990). Skin score. a semiquantitative measure of cutaneous involvement that improves prediction of prognosis in systemic sclerosis. Arthritis Rheum..

[6] Steen VD, Medsger JTA (2001). Improvement in skin thickening in systemic sclerosis associated with improved survival. Arthritis Rheum..

[7] Kang T (2014). Skin imaging in systemic sclerosis. Eur. J. Rheumatol.

[8] Cesta MF (2014). The national toxicology program web-based nonneoplastic lesion atlas:a global toxicology and pathology resource. Toxicologic Pathology.

[9] Pope JE (1995). Variability of skin scores and clinical measurements in scleroderma. J. Rheumatol.

[10] Enomoto DN, Mekkes JR, Bossuyt PM, Hoekzema R, Bos JD (1996). Quantification of cutaneous sclerosis with a skin elasticity meter in patients with generalized scleroderma. J. Am. Acad. Dermatol..

[11] Clements P (1995). Inter and intraobserver variability of total skin thickness score (modified rodnan tss) in systemic sclerosis. J. Rheumatol..

[12] Czirjak L, Foeldvari I, Muller-Ladner U (2008). Skin involvement in systemic sclerosis. Rheumatology (Oxford).

[13] Mostaço-Guidolin Leila, Rosin Nicole, Hackett Tillie-Louise (2017). Imaging Collagen in Scar Tissue: Developments in Second Harmonic Generation Microscopy for Biomedical Applications. International Journal of Molecular Sciences.

[14] Huang D (1991). Optical coherence tomography. Science.

[15] Abignano G (2013). Virtual skin biopsy by optical coherence tomography: the first quantitative imaging biomarker for scleroderma. Ann Rheum. Dis..

[16] de Boer JF, Milner TE, Nelson JS (1999). Determination of the depth-resolved stokes parameters of light backscattered from turbid media by use of polarization-sensitive optical coherence tomography. Opt. Lett..

[17] Hitzenberger C, Goetzinger E, Sticker M, Pircher M, Fercher A (2001). Measurement and imaging of birefringence and optic axis orientation by phase resolved polarization sensitive optical coherence tomography. Opt. Express.

[18] Park B, Pierce M, Cense B, de Boer J (2003). Real-time multi-functional optical coherence tomography. Opt. Express.

[19] Pires NSM (2018). Optical coherence tomography as a method for quantitative skin evaluation in systemic sclerosis. Ann. Rheum. Dis..

[20] Babalola O, Mamalis A, Lev-Tov H, Jagdeo J (2014). Optical coherence tomography (oct) of collagen in normal skin and skin fibrosis. Arch. Dermatol. Res..

[21] Ring HC (2015). Imaging of collagen deposition disorders using optical coherence tomography. J. Eur. Acad. Dermatol. Venereol..

[22] Lo WCY (2016). Longitudinal, 3d imaging of collagen remodeling in murine hypertrophic scars *in vivo* using polarization-sensitive optical frequency domain imaging. J. Invest. Dermatol..

[23] Gong P (2014). Imaging of skin birefringence for human scar assessment using polarization-sensitive optical coherence tomography aided by vascular masking. J. Biomed .Opt..

[24] Li E, Makita S, Hong YJ, Kasaragod D, Yasuno Y (2017). Three-dimensional multi-contrast imaging of *in vivo* human skin by jones matrix optical coherence tomography. Biomed. Opt. Express.

[25] Jaspers MEH, Feroldi F, Vlig M, de Boer JF, van Zuijlen PPM (2017). *In vivo* polarization-sensitive optical coherence tomography of human burn scars: birefringence quantification and correspondence with histologically determined collagen density. J. Biomed. Opt..

[26] Mogensen M, Morsy HA, Thrane L, Jemec GB (2008). Morphology and epidermal thickness of normal skin imaged by optical coherence tomography. Dermatology.

[27] Robertson K, Rees JL (2010). Variation in epidermal morphology in human skin at different body sites as measured by reflectance confocal microscopy. Acta. Derm. Venereol..

[28] Lagares David, Santos Alba, Grasberger Paula E., Liu Fei, Probst Clemens K., Rahimi Rod A., Sakai Norihiko, Kuehl Tobias, Ryan Jeremy, Bhola Patrick, Montero Joan, Kapoor Mohit, Baron Murray, Varelas Xaralabos, Tschumperlin Daniel J., Letai Anthony, Tager Andrew M. (2017). Targeted apoptosis of myofibroblasts with the BH3 mimetic ABT-263 reverses established fibrosis. Science Translational Medicine.

[29] Lakos G, Takagawa S, Varga J (2004). Animal models of scleroderma. Methods Mol. Med..

[30] Yamamoto T (1999). Animal model of sclerotic skin. i: Local injections of bleomycin induce sclerotic skin mimicking scleroderma. J. Invest. Dermatol..

[31] Junqueira LC, Montes GS, Martins JE, Joazeiro PP (1983). Dermal collagen distribution. a histochemical and ultrastructural study. Histochemistry.

[32] Zhang EZ, Vakoc BJ (2011). Polarimetry noise in fiber-based optical coherence tomography instrumentation. Opt. Express.

[33] Liang M (2015). A modified murine model of systemic sclerosis: bleomycin given by pump infusion induced skin and pulmonary inflammation and fibrosis. Lab. Invest.

[34] Fleischmajer R, Gay S, Meigel WN, Perlish JS (1978). Collagen in the cellular and fibrotic stages of scleroderma. Arthritis Rheum..

[35] Kuo WC (2007). Polarization-sensitive optical coherence tomography for imaging human atherosclerosis. Appl. Opt..

[36] Wang Z (2014). Depth-encoded all-fiber swept source polarization sensitive oct. Biomed. Opt. Express.

[37] Adams David C., Miller Alyssa J., Applegate Matthew B., Cho Josalyn L., Hamilos Daniel L., Chee Alex, Holz Jasmin A., Szabari Margit V., Hariri Lida P., Harris R. Scott, Griffith Jason W., Luster Andrew D., Medoff Benjamin D., Suter Melissa J. (2019). Quantitative assessment of airway remodelling and response to allergen in asthma. Respirology.

[38] Adams DC (2016). Birefringence microscopy platform for assessing airway smooth muscle structure and function *in vivo*. Sci. Transl. Med..

[39] Villiger M (2013). Spectral binning for mitigation of polarization mode dispersion artifacts in catheter-based optical frequency domain imaging. Opt. Express.

[40] Adams DC, Suter MJ (2018). Processing-based approach for resolving the sample optic axis in endoscopic polarization-sensitive optical coherence tomography. Opt. Express.

[41] Villiger M (2018). Optic axis mapping with catheter-based polarization-sensitive optical coherence tomography. Optica..

[42] Li Q (2018). Robust reconstruction of local optic axis orientation with fiber-based polarization-sensitive optical coherence tomography. Biomed. Opt. Express.

[43] Lagares D (2010). Endothelin 1 contributes to the effect of transforming growth factor beta1 on wound repair and skin fibrosis. Arthritis Rheum.

